# The trajectory of patterns of light and sedentary physical activity among females, ages 14-23

**DOI:** 10.1371/journal.pone.0223737

**Published:** 2019-11-06

**Authors:** Deborah A. Cohen, Bing Han, Lisa Kraus, Deborah Rohm Young

**Affiliations:** 1 Social and Economic Well Being, RAND Corporation, Santa Monica, CA, United States of America; 2 Statistics, RAND Corporation, Santa Monica, CA, United States of America; 3 Department of Research & Evaluation, Kaiser Permanente Southern California, Pasadena, CA, United States of America; Newcastle University, AUSTRALIA

## Abstract

**Purpose:**

Light physical activity (LPA) and patterns of sedentary behavior influence cardio-metabolic health independently of moderate-to-vigorous physical activity. Understanding the trajectory and determinants of these activity levels over time may provide insights relevant to public health practice.

**Methods:**

We measured a cohort of young women recruited in middle school (age 14) using accelerometry for 1 week and remeasured them in high school (age 17) and again at age 23 (n = 385). We assessed changes in LPA and patterns of sedentary behavior by hours in a day. We examined the association of social and contextual factors, including employment status, screen time, and neighborhood context with LPA and sedentary behavior patterns.

**Results:**

The amount of LPA decreased over time, while the length of LPA bouts tended to increase. Sedentary bout durations increased over time and sedentary breaks decreased. Sedentary time and bout length were correlated with internet use, rather than with TV or videogaming. Employment was associated with less sedentary time; being a student was associated with longer sedentary time and bouts.

**Conclusions:**

Because LPA and sedentary breaks can be protective for cardio-metabolic health, and the duration of sedentary bouts increase as women age from adolescence to young adulthood, worksites and college campuses should remind employees and students to take frequent activity breaks when they use computers and the internet for long stretches.

## Introduction

While moderate-to-vigorous physical activity is associated with longevity, better health outcomes and reduced prevalence of chronic diseases in adults[1, 2], many studies have also found that light physical activity (LPA)[3–5] and patterns of sedentary behavior have independent associations with health outcomes[6, 7]. Because patterns of sedentary behavior are not well-correlated with moderate-to-vigorous physical activity, those who otherwise meet physical activity guidelines may still be at risk for cardio-metabolic diseases and higher mortality, if during the rest of the day, they engage in prolonged sedentary behavior [8–10].

Patterns of activity have been described in terms of duration of uninterrupted bouts, the intensity of bouts, as well as the frequency of breaks in sedentary bouts. Breaks in sedentary behavior and short bouts of sedentary behavior have been associated with reduced cardio-metabolic risk[11] and lower BMI z- scores in children [12] as well as in adults[13, 14]. The duration of sedentary bouts and the frequency of breaks in these bouts appear to correlate with the prevalence of metabolic syndrome in both children and adults[15, 16] and with fitness in youth [17]. Adults with more LPA in long bouts had reduced cardiovascular risk compared to adults with longer bouts of sedentary behavior [18].

These physical activity patterns have physiological consequences in that they influence the transfer of glucose from the circulation into muscle cells. Reductions in physical activity over time have been associated with impaired glucose metabolism [19]. While moderate and vigorous physical activity in bouts of 10 minutes or greater are most efficient in stimulating glucose transfer into cells, physiological studies suggest that even incidental light intensity movements or resistance activities in short bouts are also effective in promoting glucose movement into muscle cells [20]. Thus, light physical activity and interruptions of sedentary behavior are important mechanisms that may reduce cardio-metabolic risk [8, 14, 21, 22]. Additional cardiovascular risks can ensue from blood viscosity and clotting factors [23, 24] and blood pressure [25–27], which are adversely affected by sedentary behaviors.

Females are particularly at risk of declining physical activity. While physical activity declines among both males and females over time [28–30], females get less physical activity than males at all time points ages and are more susceptible to conditions like osteopenia and osteoporosis from which physical activity offers protection [31–33]. However, studies have indicated that contextual factors like the design of the built environment is associated with female physical activity, so those who live in more walkable neighborhoods have lower rates of physical activity decline than those who do not [34, 35]. As youth mature, they not only leave home to live in other locations, but they pursue higher education and participate in the work force, which create settings that govern how sedentary and physically active they will be throughout the day.

The trajectory of patterns of sedentary behavior and LPA has not been well-described and there are a limited, but growing number of studies with longitudinal measurements [36–39]. This paper reports on activity patterns for a cohort of young women measured three times over a 9-year period.

## Methods

The Trial of Activity of Adolescent Girls (TAAG) was a group-randomized, controlled trial to determine if an intervention that linked schools to community organizations reduced the age-related decline in moderate-to-vigorous physical activity (MVPA) in middle school girls [40]. We enrolled 730 8th grade girls from TAAG at the Maryland field center in the Baltimore, MD/Washington DC area in the spring of 2006. Of these, 589 (81%) were re-recruited and measured during the girls’ 11th grade year (2009) using the same protocol. Six years later we re-recruited, consented, and measured 460 (63% of the original sample) when they were about 23 years of age. Complete recruitment efforts are described elsewhere [41]. The study was approved by the University of Maryland and the Kaiser Permanente Southern California Institutional Review Boards and included participant assent when the girls were younger than 18 years.

### Measures

Physical activity as the primary study outcome was measured objectively with Actigraph accelerometers (MTI model 7164). Participants wore the monitor during most waking hours for seven consecutive days. Data were collected and stored in 30-second intervals. At all measurement periods the same count thresholds were used to determine categories of physical activity intensity: Sedentary< 100/minute; Light: 100–2999; Moderate: 3000–5200; Vigorous: > 5200.[42] We excluded any days with <7.6 hours of wear time, and all participants with < 3 days of accelerometer data. Our sample size was 385, including participants with data at all 3 waves.

Bouts were defined as periods of time uninterrupted by breaks, defined as when a level of activity for at least 2 epochs of 30 seconds (1 minute) exceeded the count threshold and fell into another category of physical activity intensity. Breaks <1 minute were disregarded in the calculation of sedentary bouts. Non-wear time was identified using a series of algorithms examining outliers for dates, wear hours, and total counts, eliminating measures that were outside the study period and those too high or too low to be plausible. All activity counts were time-stamped by day and time of day. We classified sedentary behavior and LPA bouts based on their duration. We also calculated the length of the usual, median and longest bout following the methods of Bellettiere et al.[43] The usual bout length describes the bout duration at which half of the total sedentary time is accumulated, describing the central tendency of time spent in sedentary bouts [43]. In order to compare similar information by time of day across all participants in the cohort, we deleted the days with start time later than 9am or end time earlier than 9pm. This operation removed 0%, 0%, and 3% bout-level data from waves 1–3, respectively. We then examined physical activity over the same 12 hours of each day.

### Anthropometry

At age 14 and 17 years, height and weight were objectively measured using a stadiometer and calibrated scale. At age 23 years, participants self-reported their height and weight and we calculated body mass index (BMI) as kg/m2. During data collection at 17 years, we also asked girls to self-report their height and weight. Similar to other studies [44, 45], measured BMI and self-reported BMI were highly correlated (r = 0.96). Skinfold thickness of the triceps was measured on the right side of the body to the nearest millimeter to estimate body fat percentage only at ages 14 and 17.

Screen time was not measured at age 14. At ages 17 and 23, screen time was measured for internet, television, videogames, and participants were asked to estimate the time for weekdays and weekends. For each of the three categories, Computer, TV, videogames, response options were 0, <1, 1, 2, 3, 4, 5, 6 hours or more. We asked for weekdays and weekends. We transformed these into continuous variables by using 30 minutes for < 1, and for a score ranging from 0–6 for each variable. These times were summed, and weekday times were multiplied by 5, weekend times, multiplied and 2 and the total was divided by 7 to get average daily screen time.

### Geospatial calculations

Using ESRI’s ArcMap, all locations for each participant for each wave were geocoded to the street address level, and for each wave, a one-mile buffer was created. All census tract centroids that fell within the one-mile buffer were then included in the output, averaging the values for each wave. For cases where there were no tract centroids located in the one- mile buffer due to participants living in bigger tracts usually due to increased rurality, tracts whose boundaries intersected with the one-mile buffer was used to average the census data. Population density was calculated as the total population count divided by the census tract square mileage. For each survey wave, we used the respective American Community Survey (ACS)[46] tract level data. At age 14 we used the 2005–2009 5-year ACS; at age 17 we used the 2006–2010 5-year ACS and at age 23 we used the 2011–2015 5-year ACS. We obtained the walkability indices for each participant’s address from the WalkScore website.[47]

### Statistical analyses

After cleaning the data, we conducted descriptive statistics for all person-level variables over time. We also conducted graphical descriptive statistics visualizing the change in sedentary behavior and LPA patterns throughout the day.

We fitted a set of repeated measure regression models. We did not control for the initial TAAG study arm, since there were no differences in the parent TAAG study between the intervention and comparison groups. The outcomes included sedentary behavior (daily time spent in bouts with 1+ minutes and 20+ minutes; daily longest, median bouts, and usual bouts; # breaks) and LPA (daily time spent in bouts with 1+ minutes and 10+ minutes; daily longest, median, and usual bouts). The predictors included individual characteristics (race, age at baseline, employment status, student status, BMI) and neighborhood characteristics (walkability score, population density, household poverty rate). Effects of variables only collected in two waves (e.g., objectively measured BMI only measured in first two waves and employment status only measured in waves 2 and 3, ages 17 and 23) were estimated using two waves of data. Random effects were applied to adjust for within-person correlations over time. All inferences were based on robust standard errors to account for potential heteroskedasticity in some outcomes. All models were fitted by PROC MIXED in SAS 9.4.

## Results

### Population description

Table 1 describes population characteristics, sedentary behavior and LPA. Participants were of diverse backgrounds, with 48% white, 20% African American, 14% Hispanic, and 18% Asian or other race/ethnicity at baseline. The race/ethnicity of the population who were followed up at wave 3 were not different from those who dropped out after wave 2.[48] The percentage of participants who were overweight or obese increased over time, as did the average BMI. Body fat increased between ages 14 and 17. While all the participants were students at 14 and 17, only 40% were students at age 23. Full or part-time employment increased from ages 17 to 23 from 38% to 79%. The neighborhood Walk Score also increased over time, as did the neighborhood population density, reflecting more participants moving to more walkable neighborhoods over time. Average screen time, measured at 17 and 23, increased, with the largest increase in internet time, growing from 2 hours per day to nearly 4 hours per day. The average time spent playing video games dropped from about 20 minutes/day to 15 minutes/day.

**Table 1:** Population characteristics and LPA and SED behaviors by age.

	Age 14	Age 17	Age 23	p-value*
	(N = 385)	(N = 385)	(N = 385)	
Mean Age (SD)	13.8 (0.43)	16.9 (0.41)	22.9 (0.41)	< .0001
BMI (objective measure) (kg/m2)	22.3 (5.1)	23.9 (5.3)		< .0001
BMI (self-reports)(kg/m2)		23.1 (5.0)	26.0 (6.7)	< .0001
BMI categories based on CDC.gov categories				
normal/under (%) <25 or < 85^th^ %	258 (67%)	269 (70%)	214 (56%)	< .0001
Overweight (%) 25- <30; or < 95^th^ % and >85^th^ %	59 (15%)	62 (16%)	85 (22%)	< .0001
Obese (%) ≥	68 (18%)	53 (14%)	84 (22%)	< .0001
Missing (%)		1 (0%)	2 (0%)	
Percentage Body Fat	30.6 (9.2)	31.2 (7.1)	N/A	< .0001
Aggregate Activity				
# wearing days	5.2 (1.0)	5.4 (1.1)	6.8 (1.2)	< .0001
Average wearing time per day (hours) (sd)	13.9 (1.3)	13.7 (1.5)	13.9 (1.7)	0.013
% who are students (SD)	1.0 (0.0)	1.0 (0.0)	0.4 (0.5)	< .0001
% who are employed	N/A	0.38 (0.49)	0.79 (0.41)	< .0001
Smoker	0.3 (0.5)	0.6 (0.5)	0.1 (0.3)	< .0001
Walk Score of neighborhood (SD)	33.6 (23.0)	41.9 (23.1)	43.7 (28.4)	< .0001
Neighborhood population density (SD)	5,768.7 (4,218.9)	6,057.80 (4,800.0)	8,054.30 (10,944.6)	< .0001
Neighborhood % households in poverty (SD)	0.08 (0.05)	0.08 (0.05)	0.12 (0.09)	< .0001
Screen time total (hours/day) (SD)	N/A	5.35 (7.04)	6.37 (2.96)	< .0001
TV (hours/day) (SD)	N/A	1.87 (1.3)	2.37 (1.57)	< .0001
Internet (hours/day) (SD)	N/A	2.01 (1.49)	3.74 (1.73)	< .0001
Videogames (hours/day) (SD)	N/A	0.32 (0.72)	0.25 (0.83)	< .0001
***Sedentary Bout duration (minutes) mean (SE)*.**				
1+	406.6 (94.4)	417.6 (99.4)	392.8 (103.7)	< .0001
5+	253.2 (99.0)	291.5 (102.4)	282.2 (109.4)	< .0001
10+	155.6 (87.0)	199.4 (94.4)	201.9 (101.4)	< .0001
15+	104.9 (70.7)	141.3 (82.5)	151.6 (89.2)	< .0001
20+	79.6 (57.5)	105.5 (70.3)	120.2 (78.3)	< .0001
***Bout Characteristics (minutes) mean (SE)*.**				
Daily longest sedentary bout	30.4 (8.9)	36.4 (8.9)	40.3 (10.4)	< .0001
Daily median sedentary bout	1.33 (0.2)	1.54 (0.4)	1.54 (0.4)	< .0001
Daily usual sedentary bout	6.8 (2.4)	9.2 (3.1)	10.3 (4.2)	< .0001
***Number of breaks***				
30 seconds + (all bouts)	169 (27.4)	144 (27.6)	129 (27.8)	< .0001
1 min +	112 (16.9)	98 (17.0)	87 (16.2)	< .0001
***Light Physical Activity Bout duration (minutes) mean (SE)*.**				
1+	203.7 (67.2)	169.9 (70.6)	155.5 (81.4)	< .0001
2+	139.7 (58.3)	117.7 (61.3)	109.2 (72.0)	< .0001
5+	50.6 (36.3)	44.6 (39.6)	46.9 (48.5)	< .0001
10+	24.7 (20.9)	27.1 (26.2)	32.6 (35.3)	< .0001
***Light Physical activity bout characteristics (minutes) mean (SE)*.**				
Daily longest light physical activity bout	11.1 (2.9)	10.4 (3.1)	10.6 (4.2)	0.014
Daily median light physical activity bout	0.9 (0.1)	0.9(0.1)	0.9(0.2)	< .0001
Daily usual light physical activity bout	2.2 (0.6)	2. 2 (0.5)	2.3 (0.8)	0.26

* P-values are based on the overall F-test in one-way ANOVA for continuous variables, and chi-squared test for categorical variables.

**Fig 1:**
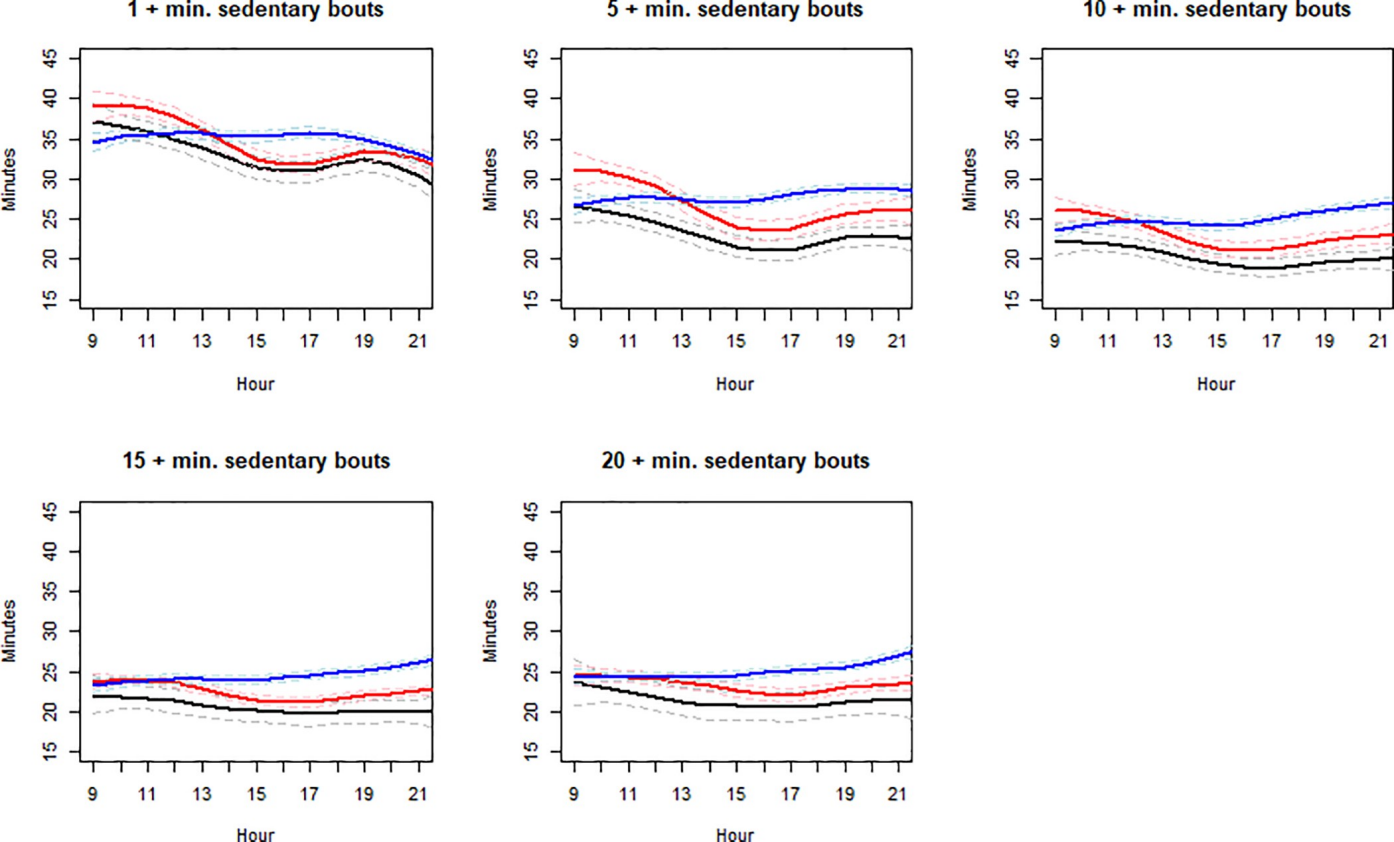
Mean sedentary minutes by bouts in different lengths for each hour between 9 am and 9 pm: Age 14 (black), Age 17 (red), Age 23 (blue). Dashed lines represent the 95% confidence band.

From ages 14–23, the total sedentary behavior did not show a clear trend (Table 1). The time spent in all sedentary bouts (1+ minute) first increased and then decreased. However, time spent in longer sedentary bouts (10+, 15+, and 20+ minutes) tended to increase continuously. Likewise, the daily longest, median, and usual bouts all increased in length from wave 1 to 3. Fig 1 illustrates the daily sedentary behavior across the hours of the day. The patterns of sedentary behavior are similar in waves 1 and 2, when participants were students in middle and high schools. Here the time spent in bouts of a certain length peaked in the morning and decreased in the afternoon and increased again in night time. In wave 3, the pattern differed. The time spent in 1+ minute sedentary bouts was flat during the daytime and decreased at night time. However, the time spent in longer bouts (e.g, 10+, or 20+ minutes), was generally increasing throughout the day. The difference in daily patterns between ages 13 and 17 and the last measurement at 23 is most obvious in longer bouts, e.g, 15+ and 20+ minutes.

**Fig 2:**
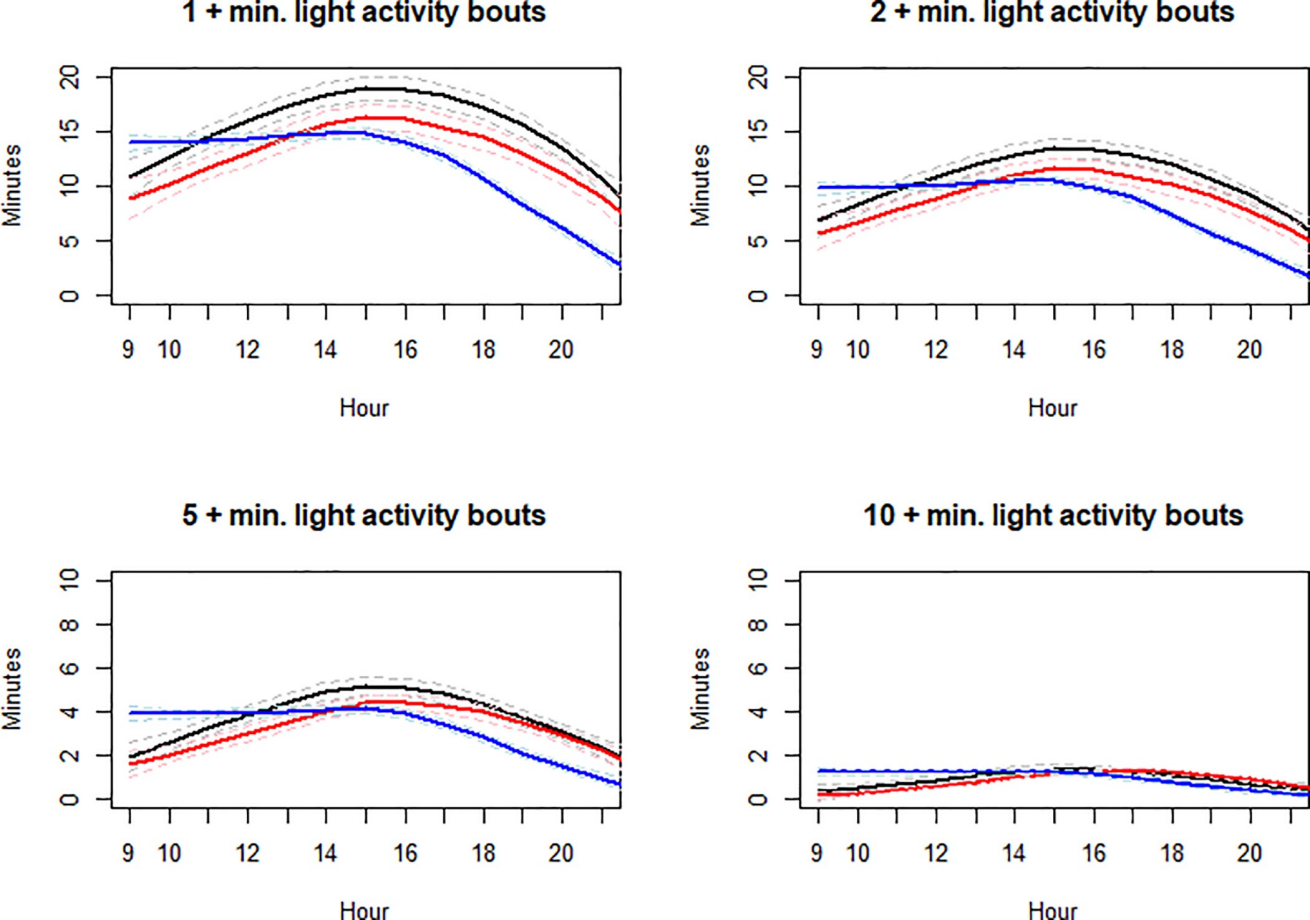
Mean light PA minutes by bouts in different lengths for each hour between 9am and 9 pm: Age 14 (black), Age 17 (red), Age 23 (blue). Dashed lines represent the 95% confidence band.

Compared with sedentary bouts, bouts of LPA were relatively uncommon, decreased over time, and totaled 60% less time than sedentary behavior (Table 1). Across all waves, most LPA time was accumulated in shorter bouts (<5 minutes). Paradoxically, the time spent in longer LPA bouts of 10 or more minutes may have increased slightly at age 23. Fig 2 shows LPA time graphed across the hours of the day. As the length of LPA bouts increase over time, the average minutes per bout decreases, because so few participants engage in long bouts of LPA. For example, at wave 3, only 40% of girls had any LPA bouts longer than 10 minutes between 3 and 4pm. At the person-day level, only 12% of person-days had recorded LPA bouts longer than 10 minutes.

LPA was almost identical at 14 and 17 except that at age 17, LPA was shifted downward in every graph, indicating less LPA time. At ages 14 and 17 all bout durations of LPA increased from 9 am to 3 pm, while at age 23, LPA was relatively constant between 9 and 3 pm. For all three waves, all bout durations of LPA decreased from 3 pm to 9 pm. Wave 3 was also unique in that before 3 pm, participants engaged in more longer LPA bouts.

### Correlates of sedentary activity bouts and breaks

After controlling for demographic variables, the strongest predictor of changes in sedentary behavior was aging, with the adjusted results the same as the non-adjusted. There was only one significant association between sedentary behavior and race/ethnicity, with African-American women having shorter median sedentary bout duration (Table 2).

**Table 2:** Models estimating associations with sedentary behavior patterns (standard error).

Effect	Daily sedentary time 1+ min bouts (minutes)	Daily sedentary time 20+ min bouts (minutes)	Daily usual bout length (minutes)	Daily median bout length (minutes)	Daily longest bout (minutes)	Daily # sedentary bouts (breaks)
Intercept	394.8 (62.8)***	62.3 (36.7)	5.1 (2.9)	1.5 (0.3)***	18.3 (9.0)*	191.6 (25.7)***
Wave 1 (reference)						
Wave 2	8.9 (4.4)*	25.3 (2.5)***	2.2 (0.2)***	0.2 (0.0)***	5.6 (0.5)***	-25.0 (1.7)***
Wave 3	-1.2 (4.7)	44.6 (3.0)***	3.3 (0.2)***	0.2 (0.0)***	9.6 (0.7)***	-39.3 (1.9)***
Day type: weekday (vs. weekend)	40.2 (2.6)***	16.2 (1.8)***	0.0 (0.0)	0.0 (0.0)	-0.1 (0.1)	-0.4 (0.2)
Age at wave 1	-1.9 (4.5)	-0.5 (2.7)	0.1 (0.2)	0.0 (0.0)	0.8 (0.7)	-1.6 (1.9)
Race: black	-8.3 (6.9)	0.6 (4.7)	-0.1 (0.3)	-0.1 (0.0)*	1.1 (0.9)	-1.4 (2.6)
Race: others	7.2 (6.9)	1.4 (4.9)	-0.1 (0.3)	0.1 (0.0)	0.3 (0.9)	1.6 (2.6)
Race: Hispanic	2.4 (6.7)	-5.2 (4.9)	-0.5 (0.4)	0.0 (0.0)	-0.9 (1.1)	5.9 (3.5)
Race: white (reference)						
Smoker: yes	1.2(4.5)	1.7 (2.8)	0.0 (0.2)	0.0 (0.0)	1.0 (0.6)	1.0 (1.7)
Walkscore	0.0 (0.1)	0.1 (0.1)	0.015 (0.006)*	0.002 (0.001)*	0.0 (0.0)	-0.1 (0.04)*
Population density/1000	-0.4 (0.4)	0.2 (0.2)	0.0 (0.0)	0.0 (0.0)	0.1 (0.1)	0.0 (0.1)
% households in poverty	49.5 (34.5)	27.2 (30.1)	-0.1 (2.5)	0.0 (0.2)	3.3 (5.9)	12.5 (15.7)
Body fat %^a^	0.3 (0.6)	0.2 (0.3)	0.0 (0.0)	0.0 (0.0)	0.1 (0.1)	-0.2 (0.2)
BMI ^a^	-1.3 (0.9)	-0.4 (0.5)	0.1 (0.0)	0.0 (0.0)	0.0 (0.1)	-0.2 (0.3)
Self-reported BMI^b^	-1.0 (0.4)*	-0.8 (0.3)*	0.0 (0.0)	0.0 (0.0)	-0.2 (0.1)**	-0.1 (0.2)
Internet time (min)^b^	0.4 (0.2)	0.6 (0.2)***	0.05 (0.01)***	0.01 (0.001)***	0.1 (0.03)**	-0.3 (0.1)**
Video game time (min)^b^	0.5 (0.3)	0.2 (0.3)	0.04 (0.02)*	0.0 (0.0)	0.2 (0.1)**	-0.1 (0.2)
TV time (min)^b^	0.2 (0.2)	0.1 (0.2)	0.0 (0.0)	0.0 (0.0)	0.0 (0.0)	0.0 (0.1)
Employment: yes ^b^	-17.4 (5.1)***	-11.3 (3.7)**	-0.5 (0.3)	-0.1 (0.0)**	-1.6 (0.8)*	2.9 (2.2)
Student: yes ^b^	16.8 (7.0)*	20.2 (4.9)***	1.2 (0.4)**	0.1 (0.0)	3.0 (1.0)**	-3.4 (2.9)

Significance level

*** p<0.001

** p<0.01

* p<0.05

^a^ Effect estimated using the first two waves

^b^ Effect estimated using the last two waves

By the third wave of data collection, the average Walk Score increased, as many participants had moved to more urbanized areas, like New York City.[35] Although the Walk Score was associated with longer usual and median sedentary bouts, the effect size was small, such that living in a highly walkable neighborhood like the Upper West Side of Manhattan (Walk Score = 97) would be associated with only 46 seconds longer duration of the usual sedentary bout length, compared to living in a much less walkable neighborhood, for example, living in a suburb like Bethesda, MD, (a Washington, DC suburb) where the Walk Score is 46.

BMI assessed at Waves 1 and 2 was not associated with any sedentary bout patterns, although self-reported BMI, assessed at Wave 3, was associated with a shorter duration of the longest sedentary bout.

Employment was associated with fewer sedentary bouts as well as a shorter duration of the median and longest sedentary bout. In contrast, being a student was associated with more sedentary time, longer bouts with the longest bout being 3 minutes longer than among participants who were not students.

Screen time was also associated with more sedentary bouts. Internet screen time was associated with sedentary breaks, but television viewing and videogaming were not. The longest sedentary bout of a young woman with 5 hours of internet time/day would be 18 minutes longer and she would have 54 fewer breaks in sedentary behavior compared to that of someone with 2 hours of internet time/ day. In addition, time spent in sedentary bouts > 20 minutes’ duration would increase by 1 hour and 48 minutes compared to the person with just 2 hours of internet time per day.

### Correlates of light physical activity

The correlates of LPA bouts were similar to those of sedentary bouts, with aging being the strongest correlate (Table 3), again with the adjusted estimates not being different from the non-adjusted ones.

**Table 3:** Models estimating associations with patterns of light physical activity (standard error).

Effect	Daily LPA time 1+ min bouts (minutes)	Daily LPA time 10+ min bouts (minutes)	Daily usual bout length (minutes)		Daily median bout length (minutes)	Daily longest bout (minutes)
Intercept	237.2 (50.7) ***	40.6 (25.1)	5.1 (2.9)		1.5(0.3)***	18.3 (9.0)*
Wave 1 (reference)						
Wave 2	-31.0 (3.3)***	1.5 (1.7)	2.2 (0.2)***		0.2(0.0)***	5.6 (0.5)***
Wave 3	-45.6 (3.7)***	5.1 (2.8)	3.3 (0.2)***		0.2(0.0)***	9.6 (0.7)***
Daytype: weekday (vs. weekend)	-12.2 (2.0)***	-5.9 (1.7)***	0.0 (0.0)		0.0(0.0)	-0.1 (0.1)
Age at wave 1	-2.1 (3.7)	-1.0 (1.8)	0.1 (0.2)		0.0(0.0)	0.8 (0.7)
Race: black	6.6 (5.1)	-1.4 (2.2)	-0.1 (0.3)		-0.07 (0.03)*	1.1 (0.9)
Race: others	-0.4 (5.5)	1.9 (2.6)	-0.1 (0.3)		0.1(0.0)	0.3 (0.9)
Race: Hispanic	5.1 (5.5)	-2.6 (2.1)	-0.5 (0.4)		0.0(0.0)	-0.9 (1.1)
Race: white (reference)						
Smoker: yes	0.7 (3.2)	-2.2 (1.4)	0.0 (0.2)		0.0(0.0)	1.0 (0.6)
Walkscore	0.0 (0.1)	0.0 (0.0)	0.02 (0.01)*		0.002 (0.001)*	0.0 (0.0)
Population Density (1,000)	0.0 (0.2)	0.1 (0.2)	0.0 (0.0)		0.0(0.0)	0.1 (0.1)
% household in poverty	21.4 (27.8)	34.4 (23.1)	-0.1 (2.5)		0.0(0.2)	3.3 (5.9)
Body fat % ^a^	-0.1 (0.4)	0.0 (0.1)	0.0 (0.0)		0.0(0.0)	0.1 (0.1)
BMI ^a^	1.2 (0.7)	-0.1 (0.2)	0.1 (0.0)		0.0(0.0)	0.0 (0.1)
Self-reported BMI ^b^	0.0 (0.4)	0.1 (0.2)	0.0 (0.0)		0.0(0.0)	-0.2 (0.1)**
Internet time (min)^b^	-0.9 (0.2)***	-0.2 (0.1)	0.005 (0.001)***		0.006 (0.001)***	0.09 (0.03)**
Video games time (min)^b^	0.1 (0.3)	0.0 (0.2)	0.05 (0.02)*		0.0(0.0)	0.2 (0.1)**
TV time (min)^b^	0.2 (0.2)	0.3 (0.1)*	0.0 (0.0)		0.0(0.0)	0.0 (0.0)
Employment: yes ^b^	20.0 (4.2)***	5.0 (1.8)**	-0.4 (0.3)		-0.09 (0.03)**	-1.7 (0.8)*
Student: yes ^b^	-11.1 (5.7)*	-5.7 (3.2)		1.2 (0.4)**	0.1 (0.0)	3.0 (1.0)**

Significance level

*** p<0.001

** p<0.01

* p<0.05

^a^ Effect estimated using the first two waves

^b^ Effect estimated using the last two waves

There were also few correlates of race/ethnicity; a difference of 4 seconds lower average median LPA bout length was found for young African-American women compared with White women. Walk Score had a positive association with LPA, such that a person living in NYC would only enjoy an average of 1 minute longer usual bout length of LPA, and 6 seconds longer median bout length compared to the same person living in Bethesda, MD.

There were no associations between LPA and measured body fat or BMI at ages 14 or 17. However, at age 23, a person who is 5 BMI units heavier (roughly 11 kgs), the longest LPA bout duration would be about 1 minute shorter than for a person 11 kgs lighter.

Associations between LPA and internet time were stronger than those of TV or videogames, although all three had some influence on LPA bouts. Internet time was associated with less total LPA, so that an additional hour of internet translates to 54 fewer minutes of daily LPA. Paradoxically, internet time also increased the usual and median LPA bout lengths as well as the longest bout of LPA, by 18 seconds, 21 seconds and 5 minutes, respectively. Videogames were associated with longer usual LPA bouts and the duration of the longest LPA bouts, while TV time was associated with more LPA time in bouts of 10 or more minutes, about 18 additional minutes per day for every additional hour of TV watched.

Employment was associated with more LPA time, but lower median and a shorter duration of the longest LPA bouts. Being a student reduced total LPA time, but increased the usual bout and the daily longest bout of LPA.

## Discussion

This study provides more evidence on increasing levels of sedentary behavior and reductions in LPA associated with young women aging from ages 14–23, and how the patterns of bouts of sedentary behavior are correlated with individual, social and contextual factors. Although our observations span age 14 to 23, it is likely that the increase in sedentary bout duration starts earlier in childhood, and may be related to school entry, as children sit at desks for a large percentage of the day.

Although it may seem obvious that being a student lends itself to sedentary behavior, it was also surprising to see that employed young women engaged in less sedentary behavior and more LPA than students. Although we consider most modern employment sedentary, it’s possible that the jobs the participants had may not have been fully sedentary. As young women, they likely would have had entry-level jobs, or may have had part-time work in service industries requiring them to be on their feet, e.g. in sales or food service.

Although studies show a strong association between neighborhood environments and moderate-to-vigorous physical activity (MVPA), other studies, including ours, find small associations between neighborhoods environments and sedentary behavior. It appears that indoor environments, such as internet-accessible places, may be more important than urban design in influencing patterns of sedentary behavior. It is possible that having wi-fi more available in outdoor settings, like public parks could influence the frequency with which people engage in active transport, even if they are sedentary at the destination [49]. Further, greater investment in public spaces, destinations, activities and events that are appealing could draw people outdoors where they may be less likely to be sedentary and/or have shorter sedentary bouts.

The domination of the internet among the screen time categories and its association with sedentary behavior and lower LPA is worrisome, particularly as the time spent on the internet increased substantially with age. Because using the internet is a task that is so engrossing, requiring interaction with the screen, we speculate that it may be difficult for people to multi-task while using it. In contrast, people can move around and even exercise while they watch TV. For example, exercise treadmills often have screens, but prevent interaction with the screen when the treadmill speed exceeds a moderate pace. Indeed, there was more longer bouts of LPA associated with TV viewing than with other types of screen time. Although previous studies have shown that TV viewing has negative consequences on moderate-to-vigorous physical activity [50, 51] for young women the internet appears to be a worse screen option than television.

However, although our study spans a longer time period, the findings of our U.S. study are quite similar to others around the world following youth over time. The EU childhood obesity project measured sedentary behavior and found that minutes of sedentary behavior increased from 299 minutes/day at age 6 to 332 min/day at age 8 to 406 min/day at age 11, while light PA also declined [38]. The UK’s Avon Longitudinal study measured youth at ages 12, 14 and 15 and showed that as sedentary behavior increased at each measurement point, light physical activity decreased commensurately [52]. Even shorter longitudinal studies, like a small Finnish study of 258 children with a 2-year follow-up showed that increases in sedentary behavior coupled with declines in MVPA were associated with increased cardiometabolic risks; however, LPA had weaker associations [36].

### Limitations

Accelerometers cannot provide information about what people are doing when they are sedentary. We don’t know whether participants were multi-tasking, watching TV, talking on the phone, playing video games, writing emails and/or studying. We did not use a time use tool that would have documented activities on an hourly basis.[53] We do have some general information about such activities that were reported by participants, however self-report, is subject to multiple biases. We narrowed our analysis to 12 hours of the day, so we could combine similar times of day among the participants. However, this means that our findings may not be generalizable to the hours before 9 am or after 9 pm. Second, several participants had short periods of missing data. Previous studies have imputed missing data as a summary of daily physical activity, and not on a minute-to-minute basis. We are unaware of techniques that could accurately impute missing sedentary bout lengths or breaks. In addition, the population was limited to a cohort of young women from the Baltimore/DC areas, so the results may not be generalizable to the general population of young women from other geographic regions of the US.

## Conclusion

The increase of longer sedentary bouts and the decline in breaks and in total light physical activity begins at a young age among females. As the girls mature, sedentary behavior appears to be strongly associated with internet time and being a student, with some protection by employment. This suggests that interventions to mitigate the increase in sedentary behavior as well as the increased duration of sedentary bouts need to be promoted in association with educational institutions, worksites and most importantly, with internet service providers. Educational institutions beyond high school should consider adopting policies that could mitigate the sedentariness promoted by study and accompanying internet use, including requiring some physical activity.

Additional strategies to reduce sedentary time should be tested. Employers also have a positive role to play. Some worksites already incorporate LPA as a part of the job, but others could promote more LPA during breaks or to interrupt other work periods that require long periods of immobility. Internet providers could automatically send messages to nudge users to take activity breaks every 20–30 minutes. A review of interventions to reduce sedentary time found that environmental interventions had the largest reductions, but worksites were able to reduce sedentary time an average of 30 minutes/day [54].

Although our measure of the walkability of the neighborhood built environments appeared to have a small association with sedentary behavior and LPA, it is still likely that environments with more opportunities for active transport could have a greater impact on both of these types of behavior.

The trends for increasing sedentary behavior with age is a significant societal challenge that will require recognition of how early the problems begin and then action on multiple levels in order to promote optimal health.

## Supporting information

S1 Table Algorithms used to calculate non-wear time. (DOCX)
