# Supplementary material for: The trajectory of patterns of light and sedentary physical activity among females, ages 14-23
Source: PLoS One. 2019 Nov 6;14(11):e0223737. doi: 10.1371/journal.pone.0223737 (PMC6834276; doi:10.1371/journal.pone.0223737)
Supplement: S1 Table — (DOCX) [file pone.0223737.s001.docx]

**S1 Table: Algorithms used to calculate non-wear time**

| **Nonwear Criteria** | **NON WEAR**  **code** | **# of days marked** |  |
| --- | --- | --- | --- |
| - Date was on or day before date monitor data was downloaded - More than 80 minutes of day had counts > 20000 cpm - Total counts > 2000000 - Total counts for the day is less than 30,000 counts OR <50,000 and date was outside wear dates provided | 1 | 362 | Within two days of download date.  IF zNhighcnt > **80** THEN outLEVEL1 = **1**;  IF totalcnt1 > **2000000** then outlevel1 = **1**;  IF totalcnt1 < **30000** then OUTlevel1 = **1**;  IF totalcnt1 < **50000** and indate = **0** then outlevel1 = **1**; |
| - Low counts, high wear hours, lots of wear in middle of night, date outside of wear dates | 2 | 120 | IF offdate ne **.** and outlevel2 = **.** then do;  If (totalcnt1 < **70000** and Date>offdate) THEN outlevel2 = **2**;  If (totalcnt1 < **70000** and Date=loadend) THEN outlevel2 = **2**;  If (totalcnt1 < **70000** and Date<ondate) THEN outlevel2 = **2**;  END;  IF outlevel2 = **.** then do;  If (totalcnt1 < **50000** and wearsimp<**360**) THEN outlevel2  = **2**;  If (totalcnt1 < **70000** and Date=loadend and pnightcnt>**30**) THEN outlevel2 = **2**;  If (totalcnt1 < **70000** and zNnonSEDcnt < **90**) THEN outlevel2  = **2**;  If (totalcnt1 < **70000** and wearsimp<**360** and pnightcnt>**24**) THEN outlevel2  = **2**;  If (totalcnt1 < **101000** and wearhr > **17.9**) THEN outlevel2  = **2**;  If (totalcnt1 < **110000** and indate = **0** and pnightcnt> **30**) THEN outlevel2 = **2**; |
| - Really high Total counts | 3 | 3 | If (totalcnt1 > **1200000**) and (outlevel2 = **.**) and (pnightcnt>**15**) THEN outlevel2 = **3**;  If (totalcnt1 > **1200000**) and (avgTcnt > **800000**) and (ID='DE556112W1') then outlevel2 = **.**; |
| - Outside of wear dates AND high percent of counts at night | 4 | 1 | If (indate = 0) and (pNIGHTcnt > 60) and (outlevel2 = .) THEN outlevel2 = 4; |
| - Wear hours > 21.5 AND high percent of counts at night | 5 | 5 | If (date = loadend) and (WEARhr > **20**) and (pnightcnt > **24**) and (outlevel2 = **.**) THEN outlevel2 = **5**;  If (WEARhr > **21.5**) and (pnightcnt > **24**) and (outlevel2 = **.**) THEN outlevel2 = **5**;  IF (wearHR > **22**) and (outlevel2 = **.**) and (indate=**0**) THEN outlevel2 = **5**; |
| - Low total counts, lower wear time, and high percent of day with zero counts. | 6 | 60 | If (WEARhr < **11**) and (zNzerocnt > **2450**) and (totalcnt1 < **100000**) and (outlevel2 = **.**) THEN outlevel2 = **6**;  IF zNzerocnt > **2300** and totalcnt1 < **100000** and (wearsimpHR < **5.5**) and (outlevel2 = **.**) THEN outlevel2 = **6**;  IF (wearsimpHR < **2**) and (INdate = **0**) and (outlevel2 = **.**) THEN outlevel2 = **6**; |
